# P-889. Therapeutic Failures of High Inoculum Infections with Cefazolin: Role of A Rapid Test and Prevalence of the Cefazolin Inoculum Effect in Methicillin-Susceptible *Staphylococcus aureus* (MSSA) Bloodstream Infections

**DOI:** 10.1093/ofid/ofae631.1080

**Published:** 2025-01-29

**Authors:** Erika Flores, Jacob Dziadula, Diana Panesso, Kavindra Singh, Lina Paola Carvajal, Sandra Rincon, Jinnethe Reyes, Asmita Ghosh, S Wesley Long, Patricia L Cernoch, Benjamin Howden, Steven Tong, José M Munita, William R Miller, Truc Cecilia Tran, Cesar A Arias

**Affiliations:** Houston Methodist Research Institute, Houston, Texas; Houston Methodist Research Institute, Houston, Texas; Houston Methodist Research Institute, Houston, Texas; Houston methodist research institute, Houston, Texas; Universidad El Bosque, Bogotá D.C., Distrito Capital de Bogota, Colombia; Universidad El Bosque, Bogotá D.C., Distrito Capital de Bogota, Colombia; Molecular Genetics and Antimicrobial Resistance Unit, Universidad El Bosque, Bogota, Distrito Capital de Bogota, Colombia; Houston Methodist Hospital, Houston, Texas; Houston Methodist, Houston, Texas; Houst, Houston, Texas; Microbiological Diagnostic Unit, Peter Doherty Institute, Melbourne, Victoria, Australia; Doherty Institute, University of Melbourne; Clínica Alemana - Universidad del Desarrollo, Santiago, Chile; Houston Methodist Research Institute, Houston, Texas; Houston Methodist Research Institute, Houston, Texas; Houston Methodist and Weill Cornell Medical College, Houston, TX

## Abstract

**Background:**

Cefazolin (CFZ) remains a key therapy for MSSA bloodstream infections (BSIs), yet some studies suggest poorer outcomes when CFZ is used in the presence of cefazolin inoculum effect (CzIE). We developed a rapid colorimetric test with nitrocefin to detect CzIE in MSSA. Here, we report the performance of the rapid test in four recurrent BSIs caused by MSSA exhibiting the CzIE. Furthermore, we assess the local prevalence of CzIE using both standard broth microdilution (BMD) and the rapid test.

**Methods:**

A chart review collected clinical data from four patients, including demographics, microbiologic features, antibiotic therapies, and clinical outcome. Patients were selected based on positive MSSA BSI after initial clearance, and availability of their index isolate. The nitrocefin-based rapid test was performed according to a published protocol. For the surveillance step, MSSA were collected from initial cultures of adult patients diagnosed with BSIs between July 2023 and April 2024 from 8 hospitals. The CzIE was assessed using BMD at standard (10^5^) and high (10^7^) inocula, with a cutoff CFZ MIC ≥ 16 μg/ml. The rapid test was performed in all isolates.

**Results:**

All four patients had deep-seated infections, including endocarditis, osteomyelitis, or device-related infections, with two patients undergoing surgical interventions. While receiving CFZ therapy, all patients successfully cleared their MSSA BSI. After initial clearance, these patients received prolonged antibiotic therapy lasting ≥ 6 weeks, with 3 of 4 patients continuing with CFZ, as part of their initial therapy. Despite this, MSSA BSI recurred between 34 and 155 days after initial hospitalization. Notably, all index isolates tested positive for CzIE via the rapid test. During the surveillance period, we collected 196 isolates, with a 40% (79/196) prevalence of the CzIE based on BMD. The rapid nitrocefin test correctly identified 72 of 79 strains exhibiting the CzIE, with 91% sensitivity and 80.3% specificity.

**Conclusion:**

These findings underscore a high prevalence of MSSA isolates exhibiting CzIE in BSIs. Patients infected with MSSA displaying CzIE may face an increased risk for recurrence when treated with CFZ. Further validation of these observations is warranted to better understand their clinical implications.

**Disclosures:**

**José M Munita, MD**, MSD: Grant/Research Support|Pfizer: Grant/Research Support **William R. Miller, M.D.**, Merck: Grant/Research Support|UptoDate: Royalties

